# Analysis of the DNA Binding Activity of BRCA1 and Its Modulation by the Tumour Suppressor p53

**DOI:** 10.1371/journal.pone.0002336

**Published:** 2008-06-11

**Authors:** Riffat Naseem, Michelle Webb

**Affiliations:** 1 Department of Medical Biochemistry and Immunology, School of Medicine, Cardiff University, Cardiff, United Kingdom; 2 Centre for Molecular Medicine, Department of Medical Genetics, School of Medicine, University of Manchester, Manchester, United Kingdom; Northwestern University, United States of America

## Abstract

**Background:**

The breast cancer susceptibility protein, BRCA1 functions to maintain the integrity of the genome. The exact mechanisms by which it does so, however, remain unclear. The ability of BRCA1 to bind directly to DNA suggests a more direct role. However, little research has been conducted to understand the functional relevance of this characteristic of BRCA1. In this study we examine the DNA substrate specificity of BRCA1 and how this may be controlled by one of its interacting partners, p53.

**Methodology/Principal Findings:**

Using competition gel retardation assays we have examined the ability of residues 230-534 of BRCA1 to discriminate between different synthetic DNA substrates that mimic those recognised by the DNA damage response i.e. four-way junction DNA, mismatch containing DNA, bulge containing DNA and linear DNA. Of those tested the highest affinity observed was for four-way junction DNA, with a 20 fold excess of each of the other synthetic DNA's unable to compete for any of the bound BRCA1 230-534. We also observed a higher affinity for C∶C and bulge containing DNA compared to linear duplex and G∶T containing DNA. BRCA1 230-534 also has interaction sites for the tumour suppressor p53 and we show that titration of this complex into the DNA binding assays significantly reduces the affinity of BRCA1 for DNA.

**Conclusions/Significance:**

In this paper we show that BRCA1 can discriminate between different types of DNA damage and we discuss the implications of this with respect to its function in DNA repair. We also show that the DNA binding activity can be inhibited by the tumour suppressor p53 and suggest that this may prevent genome destabilizing events such as HR between non-homologous sequences.

## Introduction

Cellular and environmental agents that interact with and modify the chemical structure of DNA constantly undermine the integrity of the human genome. The BRCA1 protein, which is associated with hereditary breast and ovarian cancer, acts as a scaffold that both organises and coordinates a number proteins involved in maintaining genome integrity [1], [2]. This is illustrated by the formation of a dynamic multiprotein BRCA1 Associated Genome Surveillance Complex (BASC) [2]. The BASC complex is characterised by a high number of factors that are involved in the DNA damage response, for example the mismatch repair complexes MLH1-PMS2 and MSH2–MSH6, the NBS1-MRE11-RAD50 complex which participates in pathways associated with the repair of double strand breaks and the global damage sensor ATM.

BRCA1 is a large protein of 1863 amino acids that, with the exception of an N-terminal RING domain and two tandem C-terminal BRCT domains [3], bears little homology to any other known protein. The structures of both of these domains have been determined and not only provide insights into their functional significance but also provide a structural framework for understanding cancer causing mutations [4], [5]. The RING domain has been identified in a number of proteins associated with targeted protein degradation through ubiquitination [6]. Association of the RING domains of BRCA1 with that of BARD1 results in the formation of a heterodimer which functions as an active E3 ubiquitin ligase [7]. The BRCT domain has been found in numerous proteins that function in the cellular response to DNA damage [8] and indeed this region of BRCA1 interacts with a number of proteins involved in such processes [9]–[11]. Although largely unfolded [12], [13] the central region of BRCA1 has interaction sites for a number of proteins that are associated with maintaining genome integrity (e.g. p53 [14], RAD50 [15], FANCA [16], BRCA2 [17]) and also distorted DNA structures that occur as a result of the repair of double strand breaks by homologous recombination [18], [12]. This region also contains interaction sites for RAD51 [19], which catalyses strand invasion of the homologous undamaged DNA molecule during the repair of double strand breaks by homologous recombination. BRCA1 associates directly with RAD51 and both co-localise to discrete sub-nuclear foci that redistribute to sites of DNA damage under genotoxic stress [20]. BRCA1 also co-localises with phosphorylated H2AX (γH2AX) in response to double strand breaks [21]. The formation of γH2AX foci is an early event in the DNA damage response occurring within 10 min. BRCA1 is detectable in these foci 30 min thereafter [21].

Functional support for the participation of BRCA1 in the DNA damage response is presented by studying the phenotypic outcome of targeted Brca1 deletions. For example mouse embryonic stem (ES) cells nullizygous for Brca1 show spontaneous chromosome breakage, profound genomic instability and hypersensitivity to a variety of damaging agents, e.g. γ radiation all of which suggests a defect in DNA repair [22]–[24].

The precise role BRCA1 plays in DNA repair is poorly understood. This is in part due to the lack of any identifiable functional domains. Therefore, in this present study we have used a soluble fragment of BRCA1, residues 230-534, to investigate the DNA binding specificity of BRCA1 and its interaction with the tumour suppressor p53.

## Results

### Structure specific DNA binding of BRCA1 230-534

**Figure 1:**
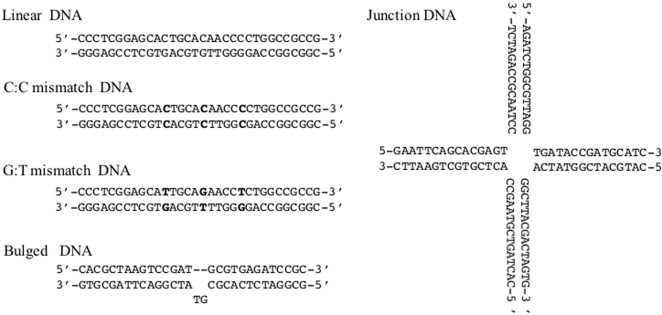
DNA substrates used to analyse the binding specificity of BRCA1 230-534.

Previously we reported the identification and characterisation of a soluble region of BRCA1 (residues 230-534) that selectively bound to four-way junction DNA when compared to linear duplex DNA [12]. The ability of BRCA1 to recognise distorted DNA structures, suggests that it may play an important role in DNA damage recognition during the repair response. Therefore, we have examined further the DNA structure selectivity of BRCA1 230-534 by assessing its relative binding affinity for a range of DNA substrates recognised in DNA repair (figure 1). In particular we have synthesised substrates that mimic those formed during the repair of double strand breaks by homologous recombination (four-way junction DNA) and those recognised by the mismatch repair machinery (G∶T, C∶C and bulge containing DNA), as BRCA1 is known to participate in these pathways [2], [25]. The design of the mismatched oligonucleotides was based upon that of Alani et al [26] and Degtyareva et al [27] as they showed that the presence of triple lesions significantly amplified the binding signal in gel retardation assays. Furthermore, clusters of mismatches are frequently left behind after recombination between two similar DNA's.

**Figure 2:**
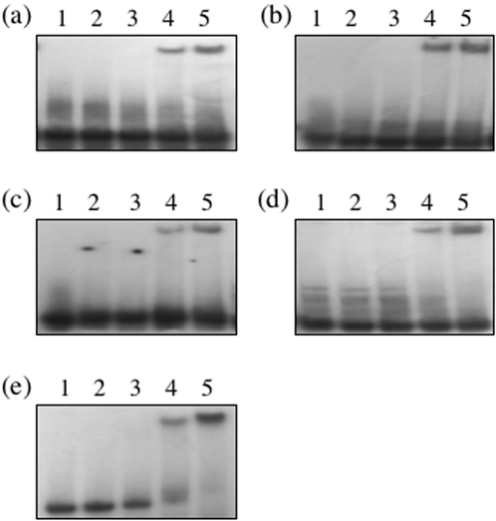
Gel retardation analysis of BRCa1 230-534 with BRCA1 was incubated with 32P labelled (a) linear duplex DNA (b) G∶T mismatch DNA (c) C∶C mismatch DNA, (d) Bulge containing DNA and (e) four-way junction DNA. In each, lane 1 contains free DNA, and lanes 2–5 contain 1,2,4 and 5 µM of BRCA1 230-534 respectively. The concentration of labelled DNA in each was 0.5 µM. Gels were visualised by autoradiography and quantified by densitometry.

**Figure 3:**
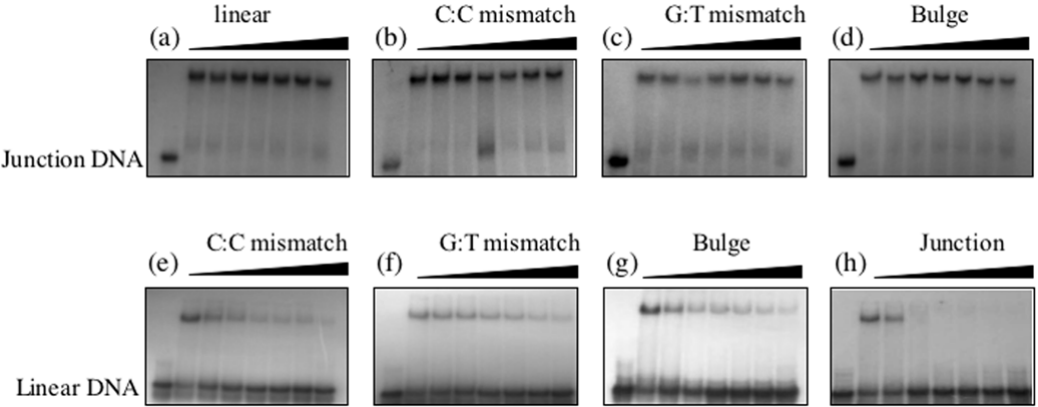
Competition gel retardation analysis of BRCA1 230-534 bound to four-way junction DNA (a–d) and linear duplex DNA (e–h). In each, lane 1 contains free DNA, lane 2 no competitor and lanes 3–8 contain 0.25, 0.5, 0.75, 1, 5 and 10 µM of each of the competitors shown. The concentration of labelled DNA and BRCA1 230-534 were 0.5 µM and 5 µM respectively. Gels were visualised by autoradiography and quantified by densitometry.

In gel retardation assays where increasing amounts of BRCA1 230-534 were titrated against each of the DNA substrates a single protein DNA complex was observed (figure 2) with initial results suggesting the highest affinity for four-way junction DNA. To study the relative binding affinities of BRCA1 230-534 in more detail competition gel retardation assays were performed in which BRCA1 230-534 was bound to radiolabelled four-way junction DNA and competed with unlabelled linear, C∶C, G∶T and bulge containing DNA. It is clear from figure 3 a–d that none of the competitors were unable to disrupt the BRCA1 230-534 four way junction complex even when present at 20 fold molar excess (lane 8). In competition assays where BRCA1 230-534 was bound to radiolabelled linear duplex DNA (figure 3 e–h) equimolar concentrations of G∶T, bulge and C∶C containing DNA reduced the amount of bound BRCA1 230-534 by 49%, 65% and 70% respectively. At equimolar concentrations a reduction of 50% would represent equal binding affinities. As expected the most effective competitor was four-way junction DNA reducing the binding of BRCA1 230-534 by 91%. These data suggest a binding preference order of four-way junction>C∶C>bulge>GT = linear duplex DNA.

### The tumour suppressor p53 interacts with the DNA binding interface of BRCA1

**Figure 4:**
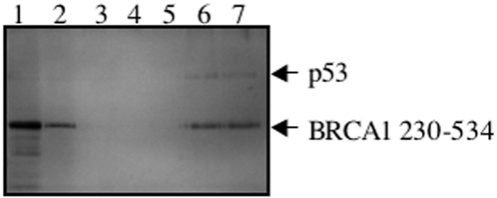
GST pull-down analysis of the interaction of BRCA1 230-534 with p53. Purified GST-p53 was absorbed onto a Glutathione Sepharose column and incubated with purified BRCA1 230-534 (lane 1). After washes with 100, 300 and 500 mM NaCl (lanes 3–5 respectively) bound BRCA1 230-534 was eluted with SDS sample buffer (lanes 6 and 7). Unbound BRCA1 230-534 was analysed in lane 2. Samples were analysed in 12% SDS PAGE gels and visualised by silver staining.

It has been reported that residues 224–500 of BRAC1 form a complex with the tumour suppressor p53 both in vivo and in vitro [14]. Therefore, we investigated the possibility that the physical association of p53 with BRCA1 may modulate its DNA binding activity. The binding of p53 to BRCA1 230-534 was first confirmed in a glutathione S-transferase (GST) pull down assay. A GST fusion of p53 was bound to glutathione sepharose beads and incubated with purified BRCA1 230-534. This resulted in the formation of a tight complex that could only be disrupted by the addition of boiling SDS (figure 4), indicating that BRCA1 230-534 not only binds to the distorted DNA structures described earlier but also p53. In the control experiment no binding of BRCA1 230-534 was observed to glutathione sepharose beads alone (data not shown).

**Figure 5:**
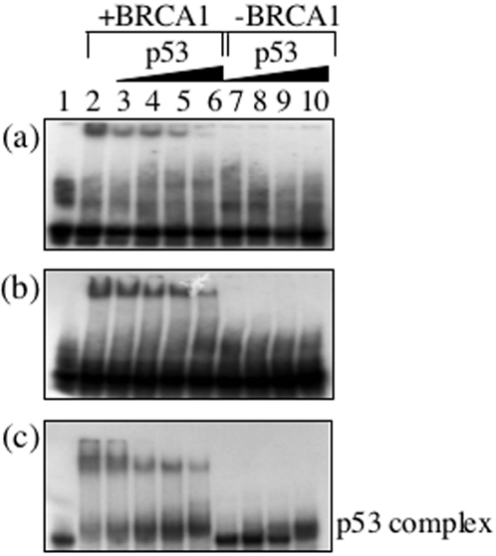
P53 reduces the DNA binding affinity of BRCA1. Titration of p53 against BRCA1 230-534 bound (a) C∶C mismatch containing DNA (b) bulge containing DNA and (c) four-way junction DNA. In all, lane 1 contains free DNA, lane 2 contains bound BRCA1230-534 with no p53 and lanes 3–6 contain 100, 200, 300 and 500 nM p53 respectively. Lanes 7–10 contain no BRCA1 230-534 and 100, 200, 300 and 500 nM p53 respectively. Samples were analysed in 6% polyacrylamide gels and visualised by autoradiography.

To determine if the interaction of p53 was able to modulate the DNA binding activity of BRCA1 it was titrated against BRCA1 230-534 bound to bulge, C∶C mismatch and four-way junction DNA (figure 5 a–c). Upon titration of p53 there is a clear reduction in the DNA binding affinity of BRCA1 230-534 to all substrates. Since no p53 binding is observed to C/C mismatch and bulged DNA this reduction in affinity can only be attributed to its interaction with BRCA1 230-534. P53 binding to four-way junction DNA has been reported previously [28] and as expected p53 bound to four-way junction DNA in the absence of BRCA1. However, a 50% reduction of BRCA1 230-534 binding is observed at a concentration where p53 is unable to bind (compare lane 4 and 8 in figure 5c). These data suggest that p53 suppresses the DNA binding activity of BRCA1.

## Discussion

BRCA1 is a large scaffolding protein that co-ordinates the appropriate protein-protein interactions and/or posttranslational modifications required for effective signal transduction upon DNA damage. This is illustrated by the formation of the multiprotein ‘repairsome’ complex BASC [2] and the requirement of BRCA1 for the ATM and ATR dependent phosphorylation of p53, c-Jun, NBS, CtIP, Smc1 and Chk 2 [1], key regulators of the DNA damage response.

In this present study we have shown that BRCA1 230-534 binds selectively to a number of structured DNA substrates recognised by the DNA damage response i.e. four-way junction DNA, bulge containing DNA and C∶C mismatch DNA. We also confirm its association with the tumour suppressor p53 as indicated by other researchers [13], [14] and show that this interaction suppresses the DNA binding activity of BRCA1.

In the repair of double strand breaks (DSB) by homologous recombination the broken DNA is resected to yield a 3′ single-strand overhang that invades a homologous donor duplex and results in the formation of a four-way junction type structure known as a Holliday junction [29]. BRCA1 230-534 shows very strong selectivity for this intermediate structure (at least 20 fold) and we propose that the integral DNA binding activity of BRCA1 is required for the effective targeting of BRCA1 mediated complexes to regions of the chromosome undergoing homologous recombination repair (HRR). During recombination mismatches and small insertions/deletion can arise as a result of strand exchange between moderately divergent (homologous) sequences [30]. The increased preference of BRCA1 for the 2 bp bulged and C∶C mismatch DNA substrates compared to linear duplex and G∶T DNA may represent a mechanism by which these lesions are sensed and repair proteins targeted to ensure the fidelity of HRR. The lack of specificity for G∶T containing DNA compared to other lesions can be explained by the association of BRCA1 with the mismatch repair protein complex MSH2–MSH6 in the BASC complex [2]. MSH2–MSH6 recognises G∶T mismatches with up 20 fold higher affinity than other mismatches, in particular C∶C [31]. Furthermore, insertion/deletions of 1 bp and >10 bp are recognised better then intermediates of 2–8 bp [31]. Therefore, the MSH2–MSH6 complex potentially targets G∶T mismatches that occur during strand exchange negating the need for this activity of BRCA1.

Although the biological outcome of inhibiting the DNA binding activity of BRCA1 needs to be clarified we can speculate that it may represent a mechanism for p53 transcription independent suppression of the repair of double strand breaks by homologous recombination [32], [33], perhaps to prevent genome destabilizing events such as HR between non-homologous sequences. Mechanisms that inhibit HR between divergent sequences while permitting recombination between identical sequences are vital as they enable repair without risking genome re-arrangement. P53 has been shown by several groups to be important in this process and while its exact role has yet to be defined it is, however, independent of MSH2 [32] a mismatch repair protein which recognises mismatches that occur during heteroduplex formation between two non-complementary DNAs. Furthermore, it cannot be explained in terms of RAD51 depletion as knockdown of p53 by RNAi has no effect on the levels of RAD51 [32]. However, there is a suggestion that p53 represses HR through physical interaction with RAD51. P53 prevents stimulation of HR by wild type RAD51 but not by a RAD51 mutant that displays reduced binding to p53 [33]. The functions of other protein involved in HR are also inhibited by interaction with p53, for instance the helicase activities of BLM and WRN [34] and the single stranded binding activity of RPA [35]. The inhibition of the DNA binding activity of BRCA1 by the direct association with p53 may, therefore, prevent HR between non-homologous DNAs ensuring the fidelity of DNA repair. Another outcome of inhibiting the DNA binding activity may be to promote apoptosis by preventing repair under conditions of extreme DNA damage. Indeed the physical association of BRCA1 and p53 when transfected into SW480 cells results in an increase in apoptosis [14]. Furthermore p53 prevents the rapid assembly of BLM-deficient BRCA1/NBS1 complexes at the stalled replication forks and induces the apoptotic response independent of its transactivation activity [36].

In summary we have shown that BRCA1 selectively targets sites of HRR potentially through its integral DNA binding activity and that this function of BRCA1 may be regulated by the tumour suppressor p53. With over 100 disease causing mutations identified in BRCA 230-534 it is easy to see how disruption of the DNA binding activity may contribute to tumour development through loss of genome integrity.

## Materials and Methods

### Purification of BRCA1 230-534 and p53

BRCA1 230-534 was over expressed from pET22b in the E. coli BL21 DE3 codon plus and purified following published procedures. The tumour suppressor p53 was over expressed as a GST fusion from pGEX2TKp53 (a kind gift from Tony Kouzaradies) in BL21 (DE3) codon plus. The p53-GST fusion was purified using Glutathione Sepharose according to the manufacturer's protocol (GE Healthcare). The experiments were carried out with the uncleaved GST fusion protein.

### Preparation of DNA substrates

Synthetic oligonucleotides were purchased from MWG Biotech. The sequences are given in Figure 1. Annealing was promoted by incubation of the appropriate oligonucleotides at 94°C for 10 min, with subsequent cooling to 4°C at a rate of 1°C/min in annealing buffer (10 mM Tris-HCl, 50 mM NaCl). The oligonucleotides were 5′-end labelled with [32P]ATP using T4 polynucleotide kinase (NEB). Unincorporated label was removed using a 1 ml G50 (GE Healthcare) spin column.

### Gel retardation assays

Gel retardation assays (10 µl) were performed in binding buffer (50 mM NaCl, 10 mM Tris-HCl (pH 8.0), 0.5 mM EDTA, 1 mM dithiothreitol, 5% glycerol) using 0.5 µM of each of the 32P-labelled DNA substrates. Samples were incubated at room temperature before analysis in 6% polyacrylamide gels. Gels were pre-electrophoresed at 150 V for 40 min, vacuum dried and visualised by autoradiography.

### Competition gel retardation assays

Binding reactions typically contained 500 nM labelled DNA, 5 µM protein 50 mM NaCl, 10 mM Tris-HCl (pH 8.0), 0.5 mM EDTA 1 mM dithiothreitol, 5% glycerol and increasing concentrations of competitor DNA in a final reaction volume of 10 µl. Competitor DNAs, 0.25, 0.5, 0.75, 1, 5 and 10 µM, were added at the start of the incubation reaction. The reactions were analysed in 6% non-denaturing polyacrylamide gels in 1× TAE buffer, pre-run for 60 min at 100 V, and electrophoresed at 150 V for ∼40 min at 4°C. Protein–DNA complexes were visualised by autoradiography.

### P53 binding assays

Purified p53 and BRCA1 230-534 were incubated with Glutathione Sepharose beads at room temperature for 1 hr in 10 mM Na phosphate (pH 7), 1 mM DTT, 1 mM EDTA, 50 mM NaCl. The beads were washed 3 times with the same buffer but containing 100 mM 300 mM and 500 mM NaCl. Bound BRCA1 230-534 was eluted by the addition of boiling SDS loading buffer. Each stage was monitored by analysis in 12% SDS polyacrylamide gels. To determine if p53 was able to modulate the DNA binding activity it was titrated at concentrations of 100 nM, 200 nM, 300 nM and 500 nM against BRCA1 230-534 bound to C∶C, bulge and four-way junction DNA in 50 mM NaCl, 10 mM Tris-HCl (pH 8.0), 0.5 mM EDTA 1 mM dithiothreitol, 5% glycerol. In all cases the concentration of labelled DNA and BRCA1 230-534 were 0.5 µM and 3 µM respectively. The reactions were analysed in 6% non-denaturing TAE polyacrylamide gels, pre-run for 60 min at 100 V, and electrophoresed at 150 V for ∼40 min at 4°C. Protein–DNA complexes were visualised by autoradiography.
